# The Professional Identity of Social Workers in Mental Health Services: A Scoping Review

**DOI:** 10.3390/ijerph20115947

**Published:** 2023-05-25

**Authors:** Harry Bark, Jeremy Dixon, Judy Laing

**Affiliations:** 1 Department of Social and Policy Sciences, University of Bath, Claverton Down, Bath BA2 7AY, UK; jd582@bath.ac.uk; 2 Law School, University of Bristol, Queen’s Road, Bristol BS8 1RJ, UK

**Keywords:** mental health social work, professional identity, mental health services

## Abstract

Recent research into the role of mental health social work has identified a need for increased critical engagement with accounts of professional role and identity. Notably, a number of studies have found that social workers struggle to articulate their role within mental health teams and services. This study aimed to identify the ways in which social workers in mental health settings defined their professional identity and role. An international scoping review utilizing Arksey and O’Malley’s method was conducted, identifying 35 papers published between 1997 and 2022. A thematic analysis grouped the findings into three predominant themes: (i) distinct social work approaches to mental health, (ii) organizational negotiations for mental health social workers, and (iii) professional negotiations for mental health social workers. These thematic findings are discussed in relation to existing research and critical perspectives, with particular emphasis on accounts of the bureaucratic and ideological functioning of professionalism in mental health services, as well as the global direction of mental health policy. This review finds that mental health social work embodies a coherent identity that aligns with international mental health policy agendas but faces significant challenges in developing and expressing this identity within mental health services.

## 1. Introduction 

Studies into the experiences of social workers in mental health settings have identified a professional role that focuses on implementing social approaches to mental health that foregrounds community and social factors alongside political and policy influences on mental distress [1,2,3,4]. Notably, Smith et al. [5] have identified a ‘dearth’ of literature on the construction and maintenance of mental health social work professional identity. The importance of this issue has also been recognized by policymakers with, for example, the Scottish Government’s *Changing Lives: Report of the 21st Century Social Work Review* notably finding that the “crisis” of social work recruitment, retention, and efficacy ‘is mainly a matter of professional identity’ [6,7]. Indeed, recent studies of English and Norwegian mental health services have found that social workers struggle to explain their role in these settings [8,9]. Professional identity, then, represents a significant point of negotiation in practice as well as being an underexplored topic in social work research, specifically in the context of mental health social work.

Professional identity is a negotiated process and phenomenon, formed through education, training, and practice [10]. Cribb and Gerwitz [11] (pp. 14–15) usefully outline professionalism as rooted in negotiations of power, with professions seeking to embody, maintain, and reproduce models of knowledge. They argue that professions hold significant social power in health and care contexts, determining notions of ‘normality’ and ‘abnormality’ in service users’ lives and making judgments on individual needs and service provisions. Professional identity, therefore, can reflect the ways in which professionals, individually and collectively, experience and assert social control through their professional epistemologies and values, and through promoting and potentially reimaging, social hierarchies and models of social functioning. Through this, the contribution of social workers to mental health services can be defined, and the professional role of social workers in these settings can be negotiated and realized. Given the global nature of this study, ‘mental health services’ is broadly defined, reflecting psychiatric, health, and social care support for service users and patients experiencing mental distress.

The form of this knowledge is, however, contested. Whilst Dixon and Richter’s [12] study finds social work to lack a distinct body of knowledge, instead drawing on core professional values and professional usefulness to services, mental health social work has, nonetheless, been understood as drawing on specific models of knowledge to offer understandings of social phenomena rooted in complex negotiations of individual and social change [13]. This occurs within a mental health field that has long been understood as a site of struggle, with the biomedical approaches of psychiatry historically dominant in services [14]. However, changes in the running of public services in liberal-democratic states from the 1970s have provided a new ideological structuring of professional power [15]. The implementation of New Public Management managerialism in the public sector, characterized by clear managerial jurisdiction, performance outcomes, and financial stringency [16], has seen bureaucracies asserting oversight of services, threatening the dominance of a single profession in health settings [17]. Nonetheless, the drive for evidence-based practice by this model of managerialism asserts a form of competition between professions and epistemologies that is broadly accepted, even within social work literature, as being best aligned with biomedical research and paradigms [5].

The practice context of mental health social work thus reflects challenging organizational, policy, and ideological terrain. Despite mental health services broadly retaining what Gould [18] (p. 17) terms an historic ‘bio-reductionist orthodoxy’, there are notable global policy approaches offered by United Nations Human Rights Council and World Health Organization reports that seek to embed the recognition of social determinants of mental health whilst also promoting social models of recovery [19,20,21]. Social work has sought to codify a professional approach to mental health that reflects such imperatives, placing it in epistemological and practice-based conflict with the predominantly individualized and pharmaceutical approaches to mental distress of psychiatric interventions [22,23]. As such, a review of existing research on mental health social work professional identity will meet an established research need and contribute to further understanding of the complex negotiations and practice experiences of mental health social work.

The completion of a scoping review, drawing on a global range of papers in a social work context, necessarily requires consideration of regional and cultural relativity [22]. As such, caution is needed when synthesizing practice perspectives, especially when directly comparing practices. Nonetheless, Spratt et al.’s [24] child protection research has identified ‘remarkable consistency’ in the issues and ideals of practice transnationally, providing a valuable framework through which to draw meaning for mental health social work practice through a scoping review. This paper thus enables the synthesis of current international perspectives on mental health social work identity and a discussion of the consequences of this knowledge for mental health and social work practice.

## 2. Materials and Methods

A scoping review provides a means of examining the extent, range, and nature of existing research, as well as identifying gaps in the literature, making it an effective approach to meeting the research aims of the study [25]. Unlike systematic reviews, scoping reviews do not seek to assess the quality of the synthesized research, instead mapping the range of existing research as an overview of a topic [26].

For this study, Arksey and O’Malley’s [25] five-stage method was adopted, enabling a rigorous search and analysis strategy that met the stated aims, objectives, and research questions of the study.

### 2.1. Stage One: Identifying the Research Question

The scoping review was guided by the overall research question of how mental health social workers understand their professional identity and role within mental health services. In answering this question, the review aimed to:

1. Provide an overview of current understandings and perspectives of social workers in mental health settings on their professional role and identity;
2. Identify research trends within the existing literature and research;
3. Identify gaps in extant research;
4. Make recommendations for future research.

### 2.2. Stage Two: Identifying Relevant Studies

Utilizing a range of sources is central to the scoping review approach and the initial search for this review, conducted in November 2022, accessed 6 electronic databases and 7 relevant peer-reviewed journals (see Table 1), yielding 2132 hits [25]. This search also drew on Mattioli et al.’s [27] discussion on reflexive supplementary approaches to keyword searches, with a ‘snowballing’ strategy adopted to also include relevant papers found in the reference lists of screened papers [28]. Through this, 3 additional relevant studies were identified.

### 2.3. Stage Three: Study Selection

Inclusion and exclusion criteria were developed to finalize the papers included in the review. Papers were required to be original, peer-reviewed, and English-language studies addressing a population of social workers in mental health practice settings, reporting on matters of professional identity and professional roles in adult mental health services. If multiple professions were reported on in a study, then distinct mental health social worker participant contribution was required. The inclusion criteria of the scoping review also required research to be qualitative but remained open to a wide range of theoretical and methodological approaches; mixed-methods studies required a distinct qualitative aspect. As the scoping review sought to gather a range of international perspectives, there was no practical cut-off date for paper inclusion to align, for example, with a particular national or statutory context. Papers published since 1997 were therefore included to provide a pragmatic 25-year window of publication synthesis.

**Figure 1:**
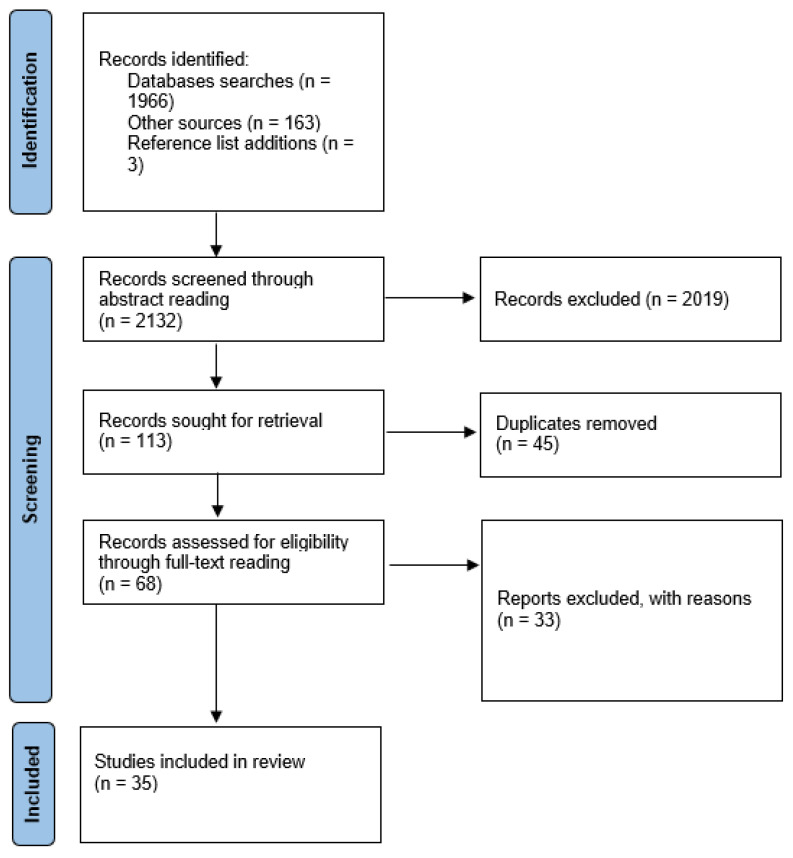
PRISMA flow chart of selection process [30].

These criteria were applied to the abstracts of the initial results with 113 papers meeting these requirements. This included the exclusion of 9 papers that were not published in English. As outlined by Badger et al. [29], an abstract cannot be assumed to represent the whole paper, and, once duplicates were removed, the remaining 68 papers were read in full; 35 papers met the criteria for inclusion in this review (see Figure 1).

### 2.4. Stage Four: Charting the Data

Drawing on Arksey and O’Malley’s [25] model, data from the selected 35 papers were extracted and charted on an Excel spreadsheet detailing the general characteristics of the studies and coding of key results that related to the research question of the scoping review.

### 2.5. Stage Five: Collating, Summarizing, and Reporting Results

This stage of the scoping review process enabled the general characteristics of the literature to be synthesized, as well as the reporting of key themes through thematic analysis (in the Results section below).

**Figure 2:**
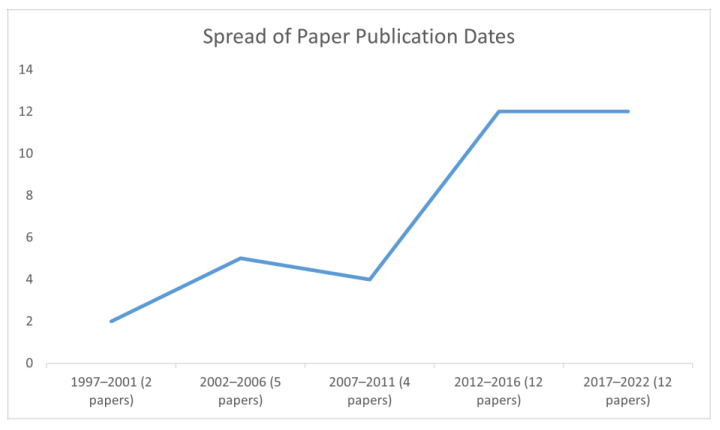
Publication trend of the reviewed literature.

The literature comprised 35 journal articles. Twenty-one nations were represented in the study. Practice perspectives from the United Kingdom were prominent (represented in 22 studies, comprised of England *n* = 17, Scotland *n* = 3, Northern Ireland, *n* = 2, Wales *n* = 1, and all the United Kingdom *n* = 1). European nations were represented in 25 studies (including the Republic of Ireland *n* = 2, and Albania, Austria, Croatia, Denmark, Norway, Slovenia, Sweden, Switzerland, and Turkey all represented once), North America in 5 studies (Canada *n* = 2, USA *n* = 2, Mexico *n* = 1), Oceania in 4 studies (Australia *n* = 3, New Zealand *n* = 1), Asia in 1 study (Hong Kong), and Africa in 1 study (South Africa). Three studies synthesized data across multiple countries. Figure 2 shows a notable increase in research activity relating to the mental health social work professional role and identity from 1997, with 69% of papers published in the last decade.

The total known sample of social workers across the dataset was 1498, with social worker participants in a single study ranging from 1 to 566. Three of the studies, consisting of 49 participants, engaged with social work students on mental health placements [5,23,31]. One paper gave distinct social worker perspectives without detailing the number of social worker participants within the 48-person sample [32]. All papers were qualitative in approach, gathering data through interviews, focus groups, and surveys. Two papers adopted mixed methods approaches which included a distinct qualitative section that was integrated into the study [33,34].

Given the global nature of these papers, the professional roles and experiences of participants were diverse. Whilst some papers provided details of the age and gender demographics of the participants, this was not widely reported. Similarly, the reporting of the career stage and professional experience level of participants was varied.

## 3. Results

A thematic analysis of the synthesized studies identified three overarching themes: (i) distinct social work approaches to mental health, (ii) organizational negotiations for mental health social workers, and (iii) professional negotiations for mental health social workers.

### 3.1. Distinctive Contributions

#### 3.1.1. Social Approaches and Values

A consistent finding across the studies was the distinct professional offering of social workers in the field of mental health through holistic, social, and rights-based approaches. Social workers emphasized the ‘widening’ of perspectives within mental health teams through their professional input, embedding an alertness to social determinants of mental health [35,36]. Social approaches to mental health were viewed as both distinct from, and an important companion to, medical understandings within services [37], with the social outlook and values of social work understood as providing a systemic perspective to health assessments of service users that may, as Morriss’ [8] study notes, be ‘invisible’ to other professionals [33]. Non-social work professionals were viewed within a number of studies as comparatively ill-equipped to embody the social approaches that underpin recovery model practice, which, despite being a contested concept, broadly seeks to reimagine the outcome of clinical recovery through a framework led by individually formed meanings and values [38,39,40]. These findings provide scope for social workers to be leaders within teams by promoting a social counter to treatment and care planning that lacks community and service user focus. A further notable aspect of the review was the identification of advocacy as a key aspect of mental health social work practice. However, this was only highlighted by student social workers. Two of the three student-orientated papers in the review foregrounded advocacy of the rights and wishes of mental health service users, both within services and on a societal basis, as a central aspect of mental health social work [23,31].

Embedding rights and social engagement in practice thus emerged as a fundamental value of mental health social work. Social workers enacted these approaches, both within and against their employing services, with accounts of professional dissatisfaction relating to mental health service provisions failing to adequately engage with social and community resources [5,9,41,42]. Indeed, both Martin’s [42] and Hurley and Kirwan’s [43] studies show social workers as recognizing social determinants of mental distress through factors such as housing and poverty issues whilst also viewing their role within services as having a far narrower remit of meeting individual mental health needs.

#### 3.1.2. Legal Roles

A predominant finding across the literature was mental health social workers identifying legal knowledge as a central and distinctive aspect of their role. This knowledge, particularly of mental health law, not only separated mental health social workers from other healthcare professionals but also from generic social work as a ‘specialized’ professional role [35]. The consideration of legal frameworks alongside varying personal, familial, and social contexts was viewed in a number of studies as a leading focus for mental health social workers, facilitating a holistic and rights-based approach to service user needs [44,45].

The emphasis on the legal role of mental health social workers was dominated by English and Welsh practice perspectives on the Approved Mental Health Professional (AMHP) role, mandated by the Mental Health Act 2007. Mental health social workers in these contexts viewed their profession as well placed to fulfil this statutory role, applying social perspectives to decisions on compulsory admission to hospitals [37,46,47]. Social workers in Morriss’ [48] study further aligned themselves with the AMHP role in recognizing an increased status and regard within mental health teams when embodying this role of legal expertise and responsibility. However, studies noted AMHP social workers as having a reduced capacity to advocate for the views of service users when assessing the need for compulsory hospital admission [49] as well as being less inclined to promote positive risk-taking, a process of reflecting on choices of action that considers the strengths and wishes of an individual [50]. Most striking was the potential for legal duties to stifle distinctive social work professional offerings, with less than a third of social worker AMHPs interviewed by Gregor [47] mentioning social approaches or models of mental health as part of their social work role. Tucker and Webber [51] also noted organizational influences on how social workers view their legal role, with those employed by English Local Authorities aligning their sense of professional identity with the statutory duties imposed on their employers, most notably through the Care Act 2014. By contrast, social workers in the study employed by the NHS did not identify the additional statutory burden of social care legislation.

Stone et al.’s [52] Europe-wide study valuably illustrates that statutory duties are not a universal aspect of mental health social work, providing a contextualizing balance to the dominance of formal legal imperatives in the British studies. Nonetheless, even in national contexts that do not have specific statutory roles that can be fulfiled by social workers, Stone et al. [52] found that practice is influenced by broader statutory and rights-based contexts, such as in foregrounding human rights in mental health services.

### 3.2. Organizational Negotiations

#### 3.2.1. Organizational Demands

In a British context, both AMHP and non-AMHP social workers identified organizational dysfunctions, such as a lack of preventative services and late referrals for support, as limiting the fulfilment of social work skills and values in practice [35,41,44,52,53,54]. There was evidence in the English context of the integration of social workers into NHS mental health teams, often through secondment from Local Authorities, as enabling social work leadership in promoting social considerations of mental health needs [33]. This arrangement, however, was also found to foster interprofessional team dynamics that hindered the abilities of social workers to fulfil social approaches in the context of medicalized mental health practices [37,55]. Uncertainty within organizational structures was also evidenced in a specific Northern Irish context, with mental health social workers noting a tension in holistically engaging with service users alongside the ‘unstated boundaries’ of religious and sectarian experiences within organizations in the context of The Troubles, manifesting in a problematic reluctance to engage with spirituality and religion as a site of meaning making [56].

Rigid organizational structures were noted in Hurley and Kirwan’s [43] study in both Irish and Canadian practice contexts, with service eligibility thresholds and ineffective interagency collaboration meaning that the skills of social workers were not fully utilized. Bureaucratic measures, such as recording statistical data, were also found to dominate practice in Khoury et al.’s [57] study of mental health social workers in Quebec, imposing an inflexible linearity to mental health recovery approaches which social workers viewed as both ineffective and undermining socially orientated recovery practice.

#### 3.2.2. Interprofessional Dynamics

Despite articulating a values-based skillset that defines professional identity, mental health social workers in the synthesized studies recognized a dominant biomedical outlook to services. This was experienced through an erasure of social perspectives in services, both through an active rejection of social approaches within teams and an overbearing medical outlook of other professionals. Social workers also perceived co-option into medicalized practices within biomedically orientated services.

A recurring theme across the review was social workers identifying an erasure of social work concepts within mental health services. Saavedra et al.’s [34] study commented on the ‘invisibilization’ of social workers in biomedically dominated mental health teams in Mexico, with the social work role reduced to that of support staff to medical colleagues. The marginalization of the very language of social work emerged in Yip’s [58] study, with social workers in Hong Kong reporting dismissive attitudes from medical colleagues towards assessments that looked beyond medical need, which were viewed as ‘unscientific’. Yip places these experiences within a framework of a biomedical hegemony within mental health services, with social workers marginalized and, ultimately, absorbed into the medical rationalization of mental healthcare. Indeed, Tucker and Webber’s [51] study finds the language of recovery and social inclusion to be strikingly absent from mental health social work practice. Similarly, Khoury et al. [57] note the co-option of the language and values of service user-led ‘recovery’ within public services into an outcome-orientated means of measuring the performance of staff and the effectiveness of services.

In many studies, social workers identified a multi-professional apathy toward integrating social approaches within mental health services. This often aligned with a numerical marginalization within teams and, at times, being ‘bottom of the hierarchy’ [32], with social workers struggling to challenge misconceptions about their role as holding a ‘single issue’ focus on matters such as housing [49,55]. The professional impotence resulting from dismissals of social approaches has resonance with mental health social worker experiences across Europe, with Stone et al. [52] noting, despite some evidence of increasingly integrated service perspectives, the continuing dominance of medical opinion and professionals in mental health teams.

Hamilton et al.’s [54] study highlights the co-option of social work into biomedical approaches within multidisciplinary teams, suggesting that, despite the integration of social workers, the dynamics of mental health teams reinforce medical paradigms rather than empower the integration of social approaches. This was experienced by social workers in a range of practice environments and cultures, varying from medical tasks, such as discussing and monitoring medication intake, to underpinning approaches to mental health within teams that were rooted in pathology and medication [43]. This was commented on by a participant in Morriss’ [8] (p. 1351) study, who described practicing as a ‘nurse with a social work body’ in such environments.

#### 3.2.3. Bridging

The present study found social workers operating flexibly within services, at times providing a professional role of advocacy on behalf of service users within teams, whilst also looking beyond their immediate organizational role to best meet mental health needs. Social workers in a number of studies identified themselves as representing a bridge between service users and other professionals, acting as mediators in times of challenging clinical treatment and support decisions [23,40]. This was also reflected in statutory practice contexts, with social workers in the AMHP role utilizing these bridging skills in discussions with bed managers, service users, and families [48].

Social workers were also likely to consider certain issues, and seek solutions, from outside of their immediate team, proactively looking beyond specific roles and responsibilities in connecting services, such as health and social care [51,59]. Whilst viewed as valuably contributing to mental health services, these bridging skills often perpetuated an ambiguity about the distinctive role of social work within teams, with social workers feeling left to ‘paper up the gaps’ within services due to their flexible outlook and approach [5] (p. 1349).

### 3.3. Professional Negotiations

#### 3.3.1. Supervision and Reflection

Reflection was understood as central to the ways social workers negotiated their professional roles and experiences, with effective supervision facilitating reflection on the wider contexts of practice and professional identity. Such opportunities were most beneficially realized when social workers were managed and supervised by social workers [35,44,58]. When practicing in mental health teams that provided supervision with a manager from another profession, these spaces were experienced as procedural and social workers took steps to seek out reflective opportunities with other social workers outside of organizational provision [5,9]. The potential for the social work role to be genericized and assimilated within mental health services was a notable consequence of such practice environments. Both Tucker and Webber’s [51] UK study and Kvaternik and Grebenc’s [60] study of mental health social workers in Slovenia noted the pressures of ‘multiple accountabilities’ to organizations, colleagues, and service users, which, without adequate understanding of the social work role by supervisors and managers, risked a dilution of social workers’ skills and effectiveness.

Social workers in Hurley and Kirwan’s [43] study across Ireland and Canada commented on the value of professional registration as a potential counter to unsupported professional development within mental health teams, with a centralized registration representing a consistent point of identity as well as development opportunities through the CPD requirements of professional bodies.

#### 3.3.2. Professional Flexibility

Within the studies, social workers were adept at recognizing tensions between their values and the expectations of organizations, prompting them to consciously assert their professional autonomy, recognize the limits to their role within organizations, and work outside of these constraints to meet their professional values and standards [51,57,60].

Social workers also demonstrated flexibility in their roles within mental health services, applying holistic relational approaches to inter-professional interactions and recognizing competing priorities and expectations in a manner that enabled leadership in collaborative casework [37,41]. Stone et al. [52] found social workers to report varying emphases on the therapeutic, statutory, and relational aspects of their roles. Such flexibility was also explored in Mallonee et al.’s [61] study as enabling the relational skills of social work to effectively adapt to the changing communication needs and possibilities heralded by the COVID-19 pandemic.

## 4. Discussion

The thematic findings of the scoping review are discussed in this section in relation to existing critical and global policy perspectives. The discussion also draws on critical accounts of the bureaucratic and ideological function of professionalism in mental health services, finding that mental health social work embodies a coherent identity that aligns with international mental health policy agendas whilst facing organizational and inter-professional challenges in expressing this identity.

### 4.1. Distinctive Contributions

#### 4.1.1. Social Approaches and Values

The synthesized literature presented a coherent and consistent account of social work as embodying a distinctive holistic and social approach to mental health. Such findings contrast with broader perspectives on interdisciplinary social work practice, in which the profession is argued as lacking its own holistic approach informed by distinct social work knowledge [62].

A claim on a distinct framework of knowledge is understood by Beddoe [63] as a necessary condition of a collective professional identity and ‘professional capital’, which, echoing Bourdieu’s [64] model of social capital as rooted in a ‘durable network’ of acquaintance and recognition, requires a codification of knowledge and the power that accompanies it. The synthesized literature points to the potential, if not always actualized, codification of dynamic social approaches to mental health as a distinctive approach of social work. As will be explored in later points of discussion, social workers face significant inter-professional, organizational, and ideological barriers to the expression of these approaches, underpinned by a global mental health research context that lacks diversified funding beyond neurobiological models [19].

#### 4.1.2. Law

Whilst the legal duties of social workers were shown in the literature to secure a professional status in mental health services, such responsibilities were also shown to problematize notions of social work professional identity [48,50,51]. There are longstanding critical and policy negotiations of this potential contradiction in practice, with Webb [65] noting that social workers may become locked into statutory duty at the expense of applying professional values and skills to their practice. Indeed, whilst AMHP social workers in a number of English and Welsh studies aligned their statutory duties with their social work knowledge and skills [37,46], the integration of other professionals (including nurses, occupational therapists, and psychologists) into the AMHP role has been critically viewed as a statutory dilution of professional identity [66]. This was echoed in the synthesized literature through discussions of risk, with studies contrasting distinctive social work approaches to ‘positive risk-taking’ [44,53], with an approach necessarily guided by coercive frameworks of risk assessment and detention by social workers within the AMHP role [47,50].

Both this review and the existing research find social workers to be attuned to personalized approaches to complexity and risk and such findings indicate the potential for social workers to adopt leadership roles in advocating for, and modelling, personalized and holistic practice [54,67]. However, there remain complex negotiations, and potential erosion of, these skills and perspectives through additional statutory responsibilities and service expectations.

### 4.2. Organizational Negotiations

#### 4.2.1. Evidence-Based Practice and Competing Professions

Despite an emphasis within the synthesized literature on English and Welsh practice experiences of organizational and structural challenges for mental health social workers, there was a consistency in the findings across a range of national contexts, with social workers negotiating distinctive professional perspectives and approaches within potentially rigid organizational arrangements and demands. Biomedical approaches to mental health have longstanding and significant prominence in services, both in terms of the number of staff who are trained in biomedical approaches to mental health, and the evidence-based practice underpinnings of services that are, broadly, best suited to quantitative and symptom reduction outcome measures [13,55]. Indeed, a prominent experience of mental health social workers across the synthesized literature was negotiations with professionals who adopt biomedical outlooks in mental health services, varying from the allocation of a limited range of tasks to interpersonal challenges rooted in a dismissal of social work approaches by medical professionals [32,34].

Such practice experiences reflect a longstanding critical tension between social work intervention and models of effectiveness guided by New Public Management principles [68] which, for Garrett [69] empowers biomedical models of practice and alienates the social worker from their guiding professional principles. Whilst traditional models of professional identity in mental health services views social work as engaged in a struggle against a prominent medical profession and its allies [70], New Public Management bureaucracy within mental health services has been identified as asserting greater control over professions and asserting an evidence-based practice framework that controls and asserts the codification of dominant knowledge and practice within services [17,71]. For Beddoe [63], services consequently become a battlefield for professions that seek to demonstrate an effectiveness that is guided by managerialist approaches and randomized controlled trial-based research that ultimately empowers pharmaceutical and psychotherapeutic interventions. A neoliberal model of performance and outcome competition within services is realized through this struggle to define roles, hierarchies, and resources [16,62] and the alignment of symptom reduction models with evidence-based practices ultimately foregrounds medical perspectives within mental health organizations.

The experience of hierarchized professions in mental health services within this review holds significant policy and ideological significance in the formation and maintenance of mental health service delivery and practice. Whilst a distinctive social work approach to mental health can be identified, there remain structural, ideological, and epistemological barriers to a consistent realization of these approaches.

#### 4.2.2. Co-Option and Erasure

As noted by Morriss [48], and evident through the findings of this review, mental health social work identity necessarily exists within services and organizations which, invariably, require formative inter-professional interactions. There is, therefore, a necessary instability to the experience of professional identity in these settings. Within biomedically dominant services, social work approaches to mental health thus operate in a system that can integrate, co-opt, and corrupt the language and logic of social work. Despite a clear expression of mental health social work identity in the review, social workers in the synthesized literature, and within the wider literature, often negotiate a challenging multi-disciplinary service context that does little to empower and promote these professional offerings and skills [4,23].

This was notably evident in the review through reported challenges in embedding service user-led recovery approaches in mental health services [72,73]. Notions of recovery and social approaches were shown to be vulnerable to assimilation into models of symptom reduction, with social workers addressing medication needs and engaging in superficial practices of ‘recovery’ that reinforced biomedical paradigms [5,57].

Beyond co-opted approaches to mental health, social workers in the synthesized literature also experienced erasure within teams, both through a dearth of social work approaches and the marginalization of the social work role itself. The language of mental health services was found to be a contested site of inter-professional struggle in a range of settings, with language linked to social factors of mental health dismissed by medical colleagues or tellingly absent from the accounts of social workers in mental health teams [51,58]. Whilst there are longstanding policy concerns relating to social workers lacking a professional voice in mental health services [74], underpinned by poorer well-being and satisfaction rates compared to other professions within multi-disciplinary teams [75], the findings point to a specific phenomenon of marginalized and suppressed social work perspectives in some mental health contexts.

Recent global conceptions of mental health services and policy have acknowledged this challenging context for social approaches to mental health, with a United Nations Human Rights Council (UNHRC) Special Rapporteur report acknowledging that:

‘the field of mental health continues to be over-medicalized and the reductionist biomedical model, with support from psychiatry and the pharmaceutical industry, dominates clinical practice, policy, research agendas, medical education and investment in mental health around the world’ [19] (pp. 5–6).

Crucially, the present study identifies power asymmetries, with biomedically orientated decision-making in mental health settings representing a barrier to the fulfilment of holistic and rights-based practices and outcomes. Such findings, in the context of this scoping review, validate the challenging circumstances of mental health social work practice and reiterate the role of social work in achieving the visions for empowering and holistic mental health service provision outlined in global documents such as the UN Special Rapporteur for Health’s report and the World Health Organization’s *Guidance on Community Mental Health Services: Promoting Person-centred and Rights-based Approaches* report [21].

### 4.3. Professional Contributions

#### Bridging and Multiple Identities

Beyond organizational challenges and negotiations for mental health social workers, a related pattern of role dilution is identified in the literature through ‘bridging’ approaches adopted by social workers, both in terms of negotiating needs and priorities between professionals and in seeking holistic solutions outside of services [23,40,51,59]. Such findings reflect an inherent flexibility in the mental health social work role. ‘Mental health social work’, conceptually, holds multiple meanings, as both individual actors placed across various settings and as a role profoundly influenced by specific organizational and contextual pressures [51,75]. Many social workers across the study identified themselves as working beyond the limitations of their organizations to seek out collaborative and creative solutions in meeting service user needs. Indeed, Oliver [76] embraces such activity through calls to assimilate the liminality of social work into professional development training as ‘boundary spanners’, operating within potentially conflicting systems and priorities in a manner that embraces negotiation and mediation.

Whilst this approach offers social work a clear role within mental health services which may not foreground social approaches to mental health, critical conceptions of professional identity note the damaging potential of uneven inter-professional collaboration, with distinctive features of a profession lost when responsibilities and practice become deeply entwined [65]. Applied to mental health social work, embracing linkages between professions may hinder potentially uncomfortable, but existentially vital, assertions of social models of mental health, ultimately providing a model for the withering away of mental health social work identity. In the context of the evidence synthesized in this review, a codifying of social work’s flexibility within mental health services through training offers a pragmatic avenue of professional clarity. Nonetheless, the pragmatism of the approach risks a pessimistic acceptance of marginalized social work perspectives on mental health, ceding the epistemological logic of mental health services and support to biomedical hegemony and constraining counter-hegemonic contestations of professional power and social approaches to mental health.

A further notable and varied experience of professional identity is evident within the literature through the role of supervision, with a clear divide between experiences of professional empowerment when management and supervision are provided by other social workers [35,45,58] compared to non-social worker professionals and management [5,9,51,60]. Furthermore, Hurley and Kirwan’s [43] finding that social workers valued holding a professional registration reflects research showing the multifaceted legitimizing role of professional registration on an organizational, inter-professional, and societal level [77]. Such factors indicate a valuable direction for professional identity expression by social workers within mental health services, drawing on Beddoe’s [63] ‘professional capital’ interpretation of networks of acquaintance and recognition. Whilst social workers consistently identify their distinctive professional skills and outlooks in the field of mental health, it is through shared connections and recognition that this professional identity is protected, enhanced, and promoted. This represents the potential of professional identity within mental health social work, creating a codified, shared, and reproducible knowledge base within services that can advocate for alternative approaches to biomedical priorities through holistic and social perspectives on mental distress and recovery.

### 4.4. Limitations and Recommendations for Future Research

Limitations to this scoping review must be noted. The implementation of inclusion and exclusion criteria, as well as the databases and journals searched, influenced the papers included in the study. This is evident in the geographical spread of synthesized studies, which were heavily weighted primarily towards English and Welsh, and European, practice contexts. The English language inclusion criteria of the review provides some explanation for this, with 9 papers excluded from the initial search (including Asian and South American studies in Korean and Portuguese) due to not meeting the language requirements, whilst resource and time constraints meant that translation was not possible. This may go some way to address the relative absence of African and Asian practice perspectives in the study (which accounted for only 6% of the synthesized papers).

Given the aims of the study, only qualitative papers were identified and synthesized. The inclusion of quantitative studies of mental health social work professional identity in future research may usefully add to knowledge about the social work role in mental health settings.

Future research into mental health social work professional identity could also beneficially explore the experiences of social workers in settings that have a greater prevalence of social workers, such as mental health social care services. There also remains a need for research (or translation of research into English) from regions that were under-represented in the study (such as Africa and Asia) to contribute towards a more representative global understanding of the mental health social work profession.

## 5. Conclusions

This paper synthesized 35 studies to consider the overarching factors in the experiences of professional identity for mental health social workers. Whilst social workers recognized their professional skills and values as offering a distinct approach within mental health teams and services, the interactions between social and medical models of mental health in these settings and, to a lesser extent, country-specific legal contexts represent a significant challenge to the realization of social work approaches. The study has placed these findings within critical accounts of professional power and influence within mental health services and global policy contexts. Through this, it can be concluded that mental health social work can be emboldened by the resonance of its professional identity with global policy approaches. The promotion of the ‘professional capital’ of mental health social work, through the development of shared connections and networks centered around the knowledge and values that social work brings to mental health services, represents a necessary component of an empowered and effective profession. There persists, however, considerable challenges and negotiations in asserting this identity in organizational and inter-professional frameworks that remain, in various ways, orientated to biomedical approaches.

## Figures and Tables

**Table 1:** Literature searches and hits.

Database	Search Terms	Hits
Scopus	(TITLE-ABS-KEY (“social work*” AND “mental health*”) AND TITLE-ABS-KEY (“professional identit*” OR values OR duties OR duty OR roles OR role OR jobs OR job OR work OR requirements) AND TITLE-ABS-KEY (experienc* OR view OR perspective* OR understanding OR perce*) AND TITLE-ABS-KEY (qualitative))	660
Web of Science	(((TS = (“social work*” AND “mental health*”)) AND TS = (“professional identit*” OR values OR duties OR duty OR roles OR role OR jobs OR job OR work OR requirements)) AND TS = (experienc* OR view OR perspective* OR understanding OR perce*)) AND TS = (qualitative)	796
IBSS	noft(“social work*” AND “mental health*”) AND noft(“professional identit*” OR values OR duties OR duty OR roles OR role OR jobs OR job OR work OR requirements) AND noft(experienc* OR view OR perspective* OR understanding OR perce*) AND noft(qualitative)	253
APA PsychNet	Abstract: “social work*” AND “mental health*” AND Abstract: “professional identit*” OR values OR duties OR duty OR roles OR role OR jobs OR job OR work OR requirements AND Abstract: experienc* OR Abstract: view OR Abstract: perspective* OR Abstract: understanding OR Abstract: perce* AND Abstract: qualitative AND Peer-Reviewed Journals only	144
Social and Policy Practice	(social work* and mental health* and (professional identit* or values or duties or duty or roles or role or jobs or job or work or requirements) and (experienc* or view or perspective* or understanding or perce*) and qualitative).ab.	87
Jstor	(Title: “social work*” AND “mental health*”) AND (All fields: “professional identit*” OR values OR duties OR duty OR roles OR role OR jobs OR job OR work OR requirements) AND (All fields: experienc* OR view OR perspective* OR understanding OR perce*) AND (All fields: qualitative)	26
Journal Title		
*British Journal of Social Work*	Title: social work* AND mental health* Abstract: professional identit* OR values OR duties OR duty OR roles OR role OR jobs OR job OR work OR requirements experienc* OR view OR perspective* OR understanding OR perce* qualitative	85
*Journal of Social Work*	(Title: social work* AND mental health*) AND (Abstract: professional identit* OR values OR duties OR duty OR roles OR role OR jobs OR job OR work OR requirements) AND (Abstract: experienc* OR view OR perspective* OR understanding OR perce*) AND (Abstract: qualitative)	30
*Journal of Social Work Practice*	[Publication Title: “social work*”] AND [Publication Title: “mental health*”] AND [[All: “professional identit*”] OR [All: values] OR [All: duties] OR [All: duty] OR [All: roles] OR [All: role] OR [All: jobs] OR [All: job] OR [All: work] OR [All: requirements]] AND [[All: experienc*] OR [All: view] OR [All: perspective*] OR [All: understanding] OR [All: perce*]] AND [All: qualitative]	5
*Social Work in Mental Health*	[Publication Title: “social work*”] AND [Publication Title: “mental health*”] AND [[All: “professional identit*”] OR [All: values] OR [All: duties] OR [All: duty] OR [All: roles] OR [All: role] OR [All: jobs] OR [All: job] OR [All: work] OR [All: requirements]] AND [[All: experienc*] OR [All: view] OR [All: perspective*] OR [All: understanding] OR [All: perce*]] AND [All: qualitative]	23
*Australian Social Work Practice*	[Publication Title: “social work*”] AND [Publication Title: “mental health*”] AND [[All: “professional identit*”] OR [All: values] OR [All: duties] OR [All: duty] OR [All: roles] OR [All: role] OR [All: jobs] OR [All: job] OR [All: work] OR [All: requirements]] AND [[All: experienc*] OR [All: view] OR [All: perspective*] OR [All: understanding] OR [All: perce*]] AND [All: qualitative]	7
*European Journal of Social Work*	[Publication Title: “social work*”] AND [Publication Title: “mental health*”] AND [[All: “professional identit*”] OR [All: values] OR [All: duties] OR [All: duty] OR [All: roles] OR [All: role] OR [All: jobs] OR [All: job] OR [All: work] OR [All: requirements]] AND [[All: experienc*] OR [All: view] OR [All: perspective*] OR [All: understanding] OR [All: perce*]] AND [All: qualitative]	4
*Social Work*	Journal: Social Work Title: social work* AND mental health* Abstract: professional identit* OR values OR duties OR duty OR roles OR role OR jobs OR job OR work OR requirements Abstract: experienc* OR view OR perspective* OR understanding OR perce* qualitative	9
‘Snowball’ Sampling		3
		Total: 2132

## Data Availability

No new data were created or analyzed in this study. Data sharing is not applicable to this article.
